# A Ready-to-Use Recombinant Yeast Two-Hybrid Assay for Thyroxine Detection

**DOI:** 10.3390/bios16080441

**Published:** 2026-08-15

**Authors:** Marius Danhausen, Sebastian Buchinger, Shimshon Belkin, Thomas Andreas Ternes

**Affiliations:** 1 Federal Institute of Hydrology (BfG), Am Mainzer Tor 1, 56068 Koblenz, Germany; danhausen@bafg.de (M.D.); ternes@bafg.de (T.A.T.); 2 The Alexander Silberman Institute of Life Sciences, The Hebrew University of Jerusalem, Edmond J. Safra Campus—Givat Ram, Jerusalem 9190401, Israel; shimshon.belkin@mail.huji.ac.il

**Keywords:** thyroid, freeze-drying, cryopreservation, long-term, field-deployable, survival rate, on-site application

## Abstract

We report a freeze-dried ready-to-use yeast thyroid screen (YTS), preserving the general dose–response characteristics of the freshly prepared counterpart. This field-deployable method reduces the assay time of the overall procedure from several days to 5 h with no requirement for sterile conditions, thus fulfilling key requirements for on-site implementation in a biosensor array. The effects of cell density and concentration of the cryoprotectant trehalose on median effective concentrations (EC_50_), limit of detection (LOD) and biosensor induction (IF) were determined and monitored over a storage period of 5 months. In addition, the impact of these parameters was monitored on the biosensor survival rate during freeze-drying and the subsequent storage process. Throughout the 5-month study, the freeze-dried recombinant yeast assay retained comparable dose–response characteristics to those of the freshly prepared counterpart, displaying median values of EC_50_ in the range of 350 nM to 550 nM and LODs in the range of 20 nM to 45 nM of the reference compound thyroxine (T4). Long-term stabilization is demonstrated using spiked (T4, 2 µM) river water and extracted wastewater effluent. After 5 months of storage, the T4-equivalent activities were 96 ± 38% and 112 ± 15% for river water and wastewater, respectively. In summary, we have successfully demonstrated a proof of principle of a field-deployable yeast thyroid screen (YTS) by using freeze-dried cells and trehalose as a cryoprotectant to achieve storability for up to 5 months at 4 °C.

## 1. Introduction

Over the last decades, the acute need for detection and quantification of endocrine-disrupting compounds (EDCs) in environmental samples has yielded several sensitive assays based on genetically engineered yeast species [1,2,3,4,5,6,7,8]. One of the approaches for these recombinant yeast assays (RYAs) is the yeast-two-hybrid (Y2H) system, in which two foreign DNA fragments are introduced into the yeast cell: a receptor able to bind agonistic ligands of interest and a reporter gene activated upon receptor binding. The expression of the reporter gene is dependent on the gene promoter driving its transcription and is induced in a concentration-dependent manner depending on the presence of receptor–ligand complexes [9,10]. This results in a measurable signal, typically detected by chromogenic [4], fluorogenic [11], luminometric [12] or electrochemical [13] methods. In comparison to bacterial-based biosensors, the eukaryotic yeast-based ones carry all the translational and post-translational tools required for mammalian gene expression. While this is also true for higher cell lines such as mammalian, insect or fish cell lines, these organisms often involve much higher cultivation, handling and maintenance costs and demands. Furthermore, such assays are often less robust to the effects of environmental matrices such as wastewater or solvents. Yeast biosensors are easy to cultivate, show high reproducibility and detection sensitivity and are relatively resilient to environmental influences. Consequently, since their first development in the 1990s, the relevance of RYAs has increased steadily as promising tools for the detection and quantification of environmental endocrine disruptors (EDCs) [8,14,15,16]. Indeed, diverse hormone-like effects such as estrogenicity [4], androgenicity [7], thyroidal activity [5], anti-estrogenicity [4] and anti-androgenicity [17] were shown to be detected by RYAs and are being utilized in ecotoxicology laboratories worldwide.

To facilitate viable sensor cell availability on demand, the continuous development of the RYA methodologies listed above has created an increased need for new approaches for cell immobilization, stabilization and preservation [18].

To address this need, we aimed to develop stable, storable and field-deployable yeast strains for RYAs, broadening the applicability of these assays while cutting overall cost, eliminating lab dependency and shortening preparation and handling times [19,20]. The yeast thyroid screen (YTS) was selected as the model RYA for applying our proposed approach.

Solutions for long-term live cell storage are mostly based on one of two approaches: preservation of the native cellular matrix by encapsulation in alginate or other polymers or storage of the cells in a desiccated state following freeze-drying (lyophilization). The former approach has been reported toenable short-term storability, reusability and automation in microfluidic systems [21,22,23], whereas the latter has been shown to support years-long viability [24,25,26]. Regardless of the method, immobilization, stabilization and preservation often result in a loss in overall biosensor performance. While many publications describe attempts at optimizing matrix embedding of bacterial and yeast, reports on freeze-dried yeast biosensors are sparse and have been limited to the estrogenic and androgenic yeast assays [27].

In this study, we characterize the suitability of freeze-drying for the yeast thyroid screen (YTS) using genetically modified *Saccharomyces cerevisiae* as the sensor strain [5] and trehalose as a cryoprotectant. While other substances such as maltose [27] or ectoine [28] can also endow cryoprotective properties, in this study we focus on trehalose due to its high water solubility and promising previous results [24,27,29]. An optimized protocol was developed for employing the lyophilized cells in a ready-to-use YTS bioassay, tested over a storage period of 5 months.

## 2. Materials and Methods

### 2.1. Strains and Media

The *Saccharomyces cerevisiae* strain used was Y190 (*MATa*, *ura3-52*, *his3-D200*, *ade2-101*, *trp1-901*, *leu2-3*, *112*, *gal4Dgal80D*, *URA3:::GAL-lacZ, cyhr2*, *LYS2:::GAL-HIS3*) containing the expression plasmids pGBT9 and pGAD424. Plasmid pGBT9 carries a part of the coding sequence of the rat TRα receptor (bp 173–461), while the pGAD424 vector carries the receptor interaction domain of the coactivator TIF2 [5]. The cells were stored at −80 °C in 30% (*v*/*v*) glycerol and cultivated overnight out of cryo-stocks at 30 °C and 200 rpm in synthetic dextrose medium (67 g/L Yeast nitrogen bases, 200 g/L D-glucose) supplemented with amino acids (146.7 mg/L adenine, 165.4 mg/L arginine, 148 mg/L histidine, 300 mg/L isoleucine, 240 mg/L lysine, 200 mg/L methionine, 500 mg/L phenylalanine, 2000 mg/L threonine, 300 mg/L tyrosine, 200 mg/L uracil, 500 mg/L valine). The yeast strain was kindly provided by Prof. Dr. Oehlmann, Goethe University Frankfurt.

### 2.2. Chemicals

All reagents were purchased from Sigma-Aldrich (St. Louis, MO, USA). Trehalose solution was prepared at a concentration of 60% *w*/*v*. A 391 µM stock solution of thyroxine was prepared in DMSO, out of which eight aqueous working solutions (199 µM to 326 nM) were prepared by eight successive 2.5-fold dilution steps, containing a final DMSO concentration of 51% (*v*/*v*). This resulted in final thyroxine assay concentrations ranging from 3.91 µM to 6.4 nM and a final DMSO assay concentration of 1%. A solution of 51% DMSO (*v*/*v*) in water without thyroxine was prepared as vehicle control.

### 2.3. Formazine Attenuation Units (FAU)

Cell densities are given in FAU. A solution of 5 g hexamethylenetetramine (C_6_H_12_N_4_) and 0.5 g hydrazine sulfate (N_2_H_6_SO_4_) in 100 mL ddH_2_O corresponds to a 4000 FAU stock solution. Out of this solution, a dilution series was prepared ranging from 800 to 25 FAU, and absorption measurements were performed at 600 nm (Abs_600_) [30]. The obtained values yield a calibration curve (Figure S1). To determine the FAU of a sample, the absorption of the sample is measured at 600 nm and corrected by the corresponding medium blank. The corrected value is then compared to the calibration curve, and the FAU are calculated.

### 2.4. Recombinant Yeast Assays (RYAs)

An aliquot (5 mL) of synthetic dextrose medium was inoculated with 300 µL of a cryo-stock and incubated at 30 °C and 200 rpm shaking for 20 h. The following day, the cell culture density at 600 nm was adjusted to 30 FAU for the RYAs performed with freshly cultivated yeast. Aliquots of 50 µL were transferred into a 96-well plate, and 1 µL of each dilution of the thyroxine dilution series was added. The remaining wells were filled with incubation medium to serve as a procedure blank. Plates were incubated at 30 °C for 18 h, following which Abs_600_ was measured with a Tecan Infinite 200 Pro plate reader (Tecan, Maennedorf, Switzerland; device set to 5 flashes and 0 ms of waiting time). Activity was assayed in *lacZ* buffer (10.67 g Na_2_HPO_4_·H_2_O, 0.75 g KCl, 0.25 g MgSO_4_·7 H_2_O, 1.0 g sodium dodecyl sulfate (SDS) in 1 L ddH_2_O) containing 0.4 g/L chlorophenol red-β-D-galactopyranoside (CPRG), 2 g/L cysteine and 0.383 g/L lyticase (652 units). Exactly 80 µL was added to every well following incubation at 30 °C for 1 h at 200 rpm. Subsequently, the absorption at 578 nm (Abs_578_) was determined to quantify the activity of the reporter enzyme. All experiments were performed at least in independent duplicates containing three technical replicates each.

RYAs performed to test the influence of medium viscosity were prepared by mixing 25 µL of a 300 FAU cell suspension in a 2× medium with 25 µL of water or a 60% (*w*/*v*) trehalose solution and measured after exposure times of 2, 4 and 18 h.

### 2.5. Freeze-Drying Protocol

The following section describes the optimized freeze-drying conditions developed within the current study. Deviating conditions with respect to cell density and trehalose concentration are described in the respective figure legends.

Cell suspension aliquots (25 µL, 1000 FAU) were mixed in 96-well plates with 25 µL of a 40% (*w*/*v*) trehalose solution to a final trehalose concentration of 20% (*w*/*v*) and a final cell density of 500 FAU. The 96-well plates were frozen at −80 °C for 2 h and subsequently freeze-dried overnight at 1.6 mbar and −78 °C using a Christ™ Gamma 1–16 LSC freeze dryer (Martin Christ, Osterode, Germany). The plates were reconstituted immediately after freeze-drying at 30 °C for 2 h in 50 µL of cell medium before induction with 1 µL of thyroxine solution. After an additional 2 h incubation, Abs_600_ was measured with a plate reader. The further procedure was performed as described above.

### 2.6. Determination of Cell Viability

Survival of the freeze-dried yeast cells was determined by counting the number of colony-forming units (CFUs) on 2% agar dextrose growth medium. One microliter of the fresh yeast suspension with a cell density of either 1000, 2000 or 4000 FAU was diluted 2,000,000-fold. Aliquots (20 µL) of this suspension were evenly spread on agar plates and incubated overnight. The number of CFUs was counted and compared to the number of CFUs corresponding to the respective yeast suspension yielded after freeze-drying. For this, cells were reconstituted in 50 µL of medium. The resulting cell suspensions from three separate vehicle control wells were pooled. This suspension was diluted 10,000-fold, and 20 µL was incubated on plates overnight. The survival rate after freeze-drying was expressed as a percentage of the corresponding fresh yeast CFUs under consideration of the respective dilution factors. All experiments were performed in duplicates.

### 2.7. Testing of Spiked Environmental Samples

To test the effect of environmental matrices on the standard and freeze-dried RYAs, natural Rhine water was collected. Additionally, a 1000-fold concentrated wastewater effluent from a municipal wastewater-treatment plant (WWTP) was prepared by solid-phase extraction (SPE) using Oasis HLB cartridges, conditioned with 3 × 2 mL methanol and 4 × 2 mL ddH_2_O. After loading the water sample, cartridges were dried and eluted with 10 mL methanol. To keep results comparable to further experiments, the solvent was changed from methanol to DMSO by evaporation. After testing both matrices negative for their native thyroid activity, the Rhine water sample was spiked with T4 to a final concentration of 2.5 µM. From this solution, a dilution series (2-fold dilution steps, 2.5 µM to 20 nM) was prepared. For exposure to the standard RYAs, 40 µL of the Rhine water dilution series was mixed with 10 µL of 5× growth medium, resulting in final T4 well concentrations of 2 µM to 16 nM. In the case of the freeze-dried RYAs, the same mixture was used to directly reconstitute the cells after freeze-drying. The WWTP extract was diluted to a relative enrichment factor of 30x and spiked with T4 to a final concentration of 10 µM. A corresponding dilution series was prepared ranging from 10 µM to 80 nM. For exposure to the RYAs, 10 µL of the WWTP extract dilution series was mixed with 40 µL of 1.25× growth medium, resulting in a final relative enrichment factor of 6x and a final T4 well concentration of 2 µM to 16 nM. All experiments were performed in duplicate with three technical replicates each. The thyroxine reference was performed in technical duplicates.

### 2.8. Long-Term Stability

Replicates of the freeze-dried cells prepared as described above were stored in vacuum-sealed bags at 4 °C and reconstituted as described after 3 days, 7 days, 3 weeks (21 days), 8 weeks (60 days) and 5 months (150 days). All plates were prepared out of one cell culture and freeze-dried as described in Section 2.5 within 24 h in three batches, due to the maximum loading capacity of the freeze dryer. No duplicates were freeze-dried in the same batch. The calculated IF, EC_50_, LOD and CFUs, as well as the long-term effects of cell density and trehalose concentration, were compared to the results obtained with fresh RYAs and RYAs with cells immediately reconstituted after freeze-drying. Biosensor performance following long-term storage was also determined by testing the spiked environmental matrices after 1, 7, 21 and 150 days.

### 2.9. Data Analysis

To simplify and to condense experimental results, effects are displayed using the induction factor (IF) calculated as the mean Abs_578_ divided by the mean Abs_578_ of the corresponding negative control (nc) of both plates with technical triplicates each (Equation (1)):

(1) IF = Abs_578_/Abs_578,nc_

The error of the IF was calculated according to Gaussian error propagation on the basis of the standard deviation measured for each concentration level and negative control. A four-parameter sigmoidal function (Equation (2)) was fitted with the *drm* model in *R* (version 2.5-12) to the dose-dependent IF values to determine the corresponding EC_50_ values. *a* displays the minimum value, *b* the maximum value, *c* the turning point, *d* the steepness and *x* the T4 well concentration corresponding to the measured induction *y* [31]. The LOD is determined at min + 3×σ_nc_.

(2) (a − b)/(1 + (x/c)^d^) = y

Regarding the testing of environmental matrices, the reference dose–response data was processed the same way. The IF values of the samples were calculated and compared to the fitted four-parameter sigmoidal function, while considering the dilution factor, to obtain T4-equivalent well concentrations for each of the eight dilutions. The T4-equivalent well concentrations of dilution steps 3 to 6 were averaged, since the induction in this range corresponded to the steepest part of the dose–response curves, resulting in the highest accuracy.

The z-factor is a measure of statistical effect size and was calculated as denoted elsewhere [32]. Values above 0.5 display a statistically reliable assay, while z-factors between 0 and 0.5 are classified as an assay of marginal quality. Values below 0 indicate an assay response with no significant difference from the negative control.

Determination of the initial cell survival rates, as well as the corresponding decay constants, was performed by fitting a model for exponential decay to the survival rates observed (Equation (3)). N_0_ displays the starting value at timepoint zero, and k is the decay constant.

(3) N(t) = N_0_ × e^(−kt)^

Significance tests were performed using the compromise function in G*Power 3.1.9.7 to calculate the α error probability, which is reported together with the respective observation (*p*-value) [33]. If not stated otherwise, errors are given as standard deviations (SD).

## 3. Results

### 3.1. Selection of Freeze-Drying Conditions

In order to identify the optimal combination of cryoprotectant concentration and cell density, a matrix comprising densities from 25 to 4000 FAU and trehalose concentrations of 0, 10, 20 and 30% was tested. Standard RYAs are performed at cell densities of 25 to 100 FAU, but loss of cell viability during the freeze-drying process may necessitate higher cell densities [24,27]. Since the tested combinations were prepared by mixing trehalose solution and yeast suspension at a 1:1 ratio, 30% was the maximal trehalose concentration examined, in view of this sugar’s solubility limit.

**Figure 1:**
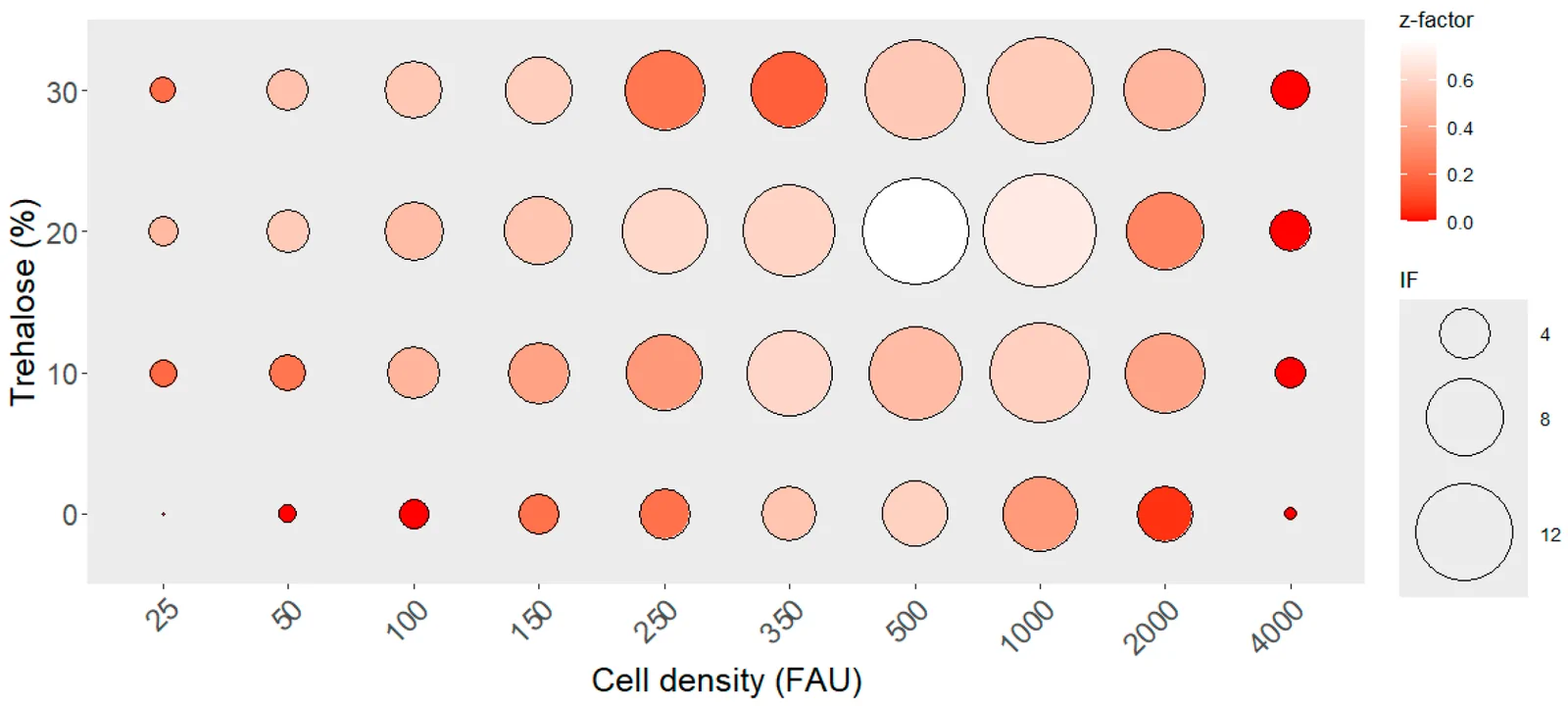
Dependence of cell density and concentration of cryoprotectant on the IF and z-factor. The cells were directly reconstituted after freeze-drying and induced with 1 µL of 0.25 µM T4. Abs_578_ was measured, and the IF values, as well as the z-factors, were calculated (n = 6). All z-factors of zero or below are displayed in red.

Figure 1 shows that the induction intensity increases with increasing cell density, with a maximum at cell densities of 500 and 1000 FAU, above which a reduction in the IF occurred. The addition of trehalose resulted in increased induction, but the IF values were virtually similar for the three different trehalose concentrations tested. In contrast, the trehalose concentration impacted the z-factor, with the highest values obtained with a trehalose concentration of 20%. After the freeze-drying process, only cell densities of 350 to 1000 FAU yielded IF values higher than 10 and are thus comparable to induction factors of 20 to 30 usually observed for such assays. Due to the very low standard deviations observed for cell densities of 50 to 1000 FAU (see Table S1), the calculated z-factors are above 0.4 when stabilized with 20% or 30% trehalose, thus classifying these conditions as suitable for dose–response analysis. In contrast to the general trend, the z-factors determined for cell densities of 250 and 350 FAU in combination with a trehalose concentration of 30% led to high standard deviations between physical and technical replicates, resulting in low z-factors. The highest IF was achieved with 20% trehalose and a cell density of 1000 FAU, while the highest z-factor was achieved with 20% trehalose and a cell density of 500 FAU. Since the difference in z-factor outweighs the difference in IF, the best overall assay performance was achieved with 20% trehalose and a cell density of 500 FAU.

### 3.2. Effect of Freeze-Drying on Dose–Response Characteristics and Cell Performance

After the initial screening for suitable freeze-drying conditions, full concentration response curves for the reference compound T4 were characterized to derive performance characteristics such as EC_50_ and LOD in comparison to freshly prepared yeast cells.

**Figure 2:**
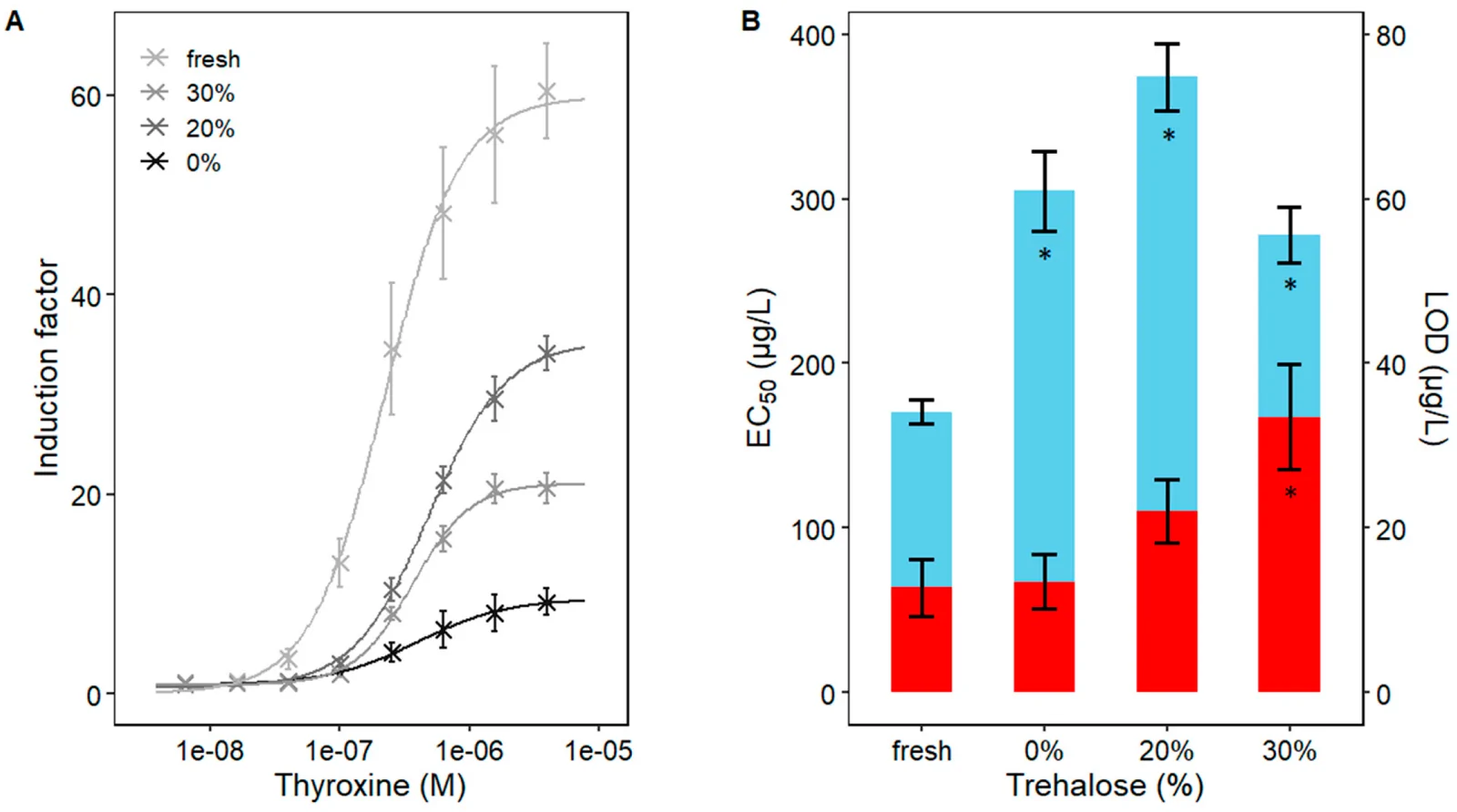
Effect of freeze-drying with and without cryoprotectant on the IF values and detection sensitivity compared to the standard RYA. Cell suspensions with 1000 FAU were freeze-dried with 0%, 20% and 30% trehalose (*w*/*v*) and immediately reconstituted prior to induction with T4. Abs_578_ was measured, and the IF values were calculated (n = 2). The resulting concentration-dependent IF values are compared against historical dose–response data of the standard RYA at 30 FAU. Four-parameter sigmoidal functions were fitted (**A**) from which the EC_50_ (blue) and the LODs (red) were determined (**B**). Asterisk (*) shows a significant (*p* < 0.01 for EC_50_; *p* < 0.05 for LOD) increase compared to the freshly prepared cells.

The dose-dependent response of freeze-dried RYAs with a cell density of 1000 FAU directly reconstituted after freeze-drying was compared to the response of the standard RYA using fresh cells (Figure 2). While the maximal induction intensity was reduced following the freeze-drying process, with absolute values dropping from 60.4 to 34.1, 20.6 and 9.2 in the presence of 20%, 30% or 0% trehalose, respectively, the general dose–response characteristics were not impacted by the process. The standard RYA showed an EC_50_ of 171 ± 8 µg/L and a LOD of 13 ± 4 µg/L. For the freeze-dried variants, corresponding values of EC_50_ = 278 ± 17 µg/L and LOD = 34 ± 7 µg/L (30% trehalose), EC_50_ = 374 ± 20 µg/L and LOD = 22 ± 4 µg/L (20% trehalose) and EC_50_ = 305 ± 25 µg/L and LOD 14 ± 4 µg/L for the unstabilized variant were determined, showing a significant loss in sensitivity due to an increased EC_50_ (*p* < 0.01) and increased LOD (*p* < 0.05; significant only for 30% trehalose) when compared to the sensitivity of the freshly prepared RYA (Figure 2).

### 3.3. Effect of Long-Term Storage on Sensor Performance

**Figure 3:**
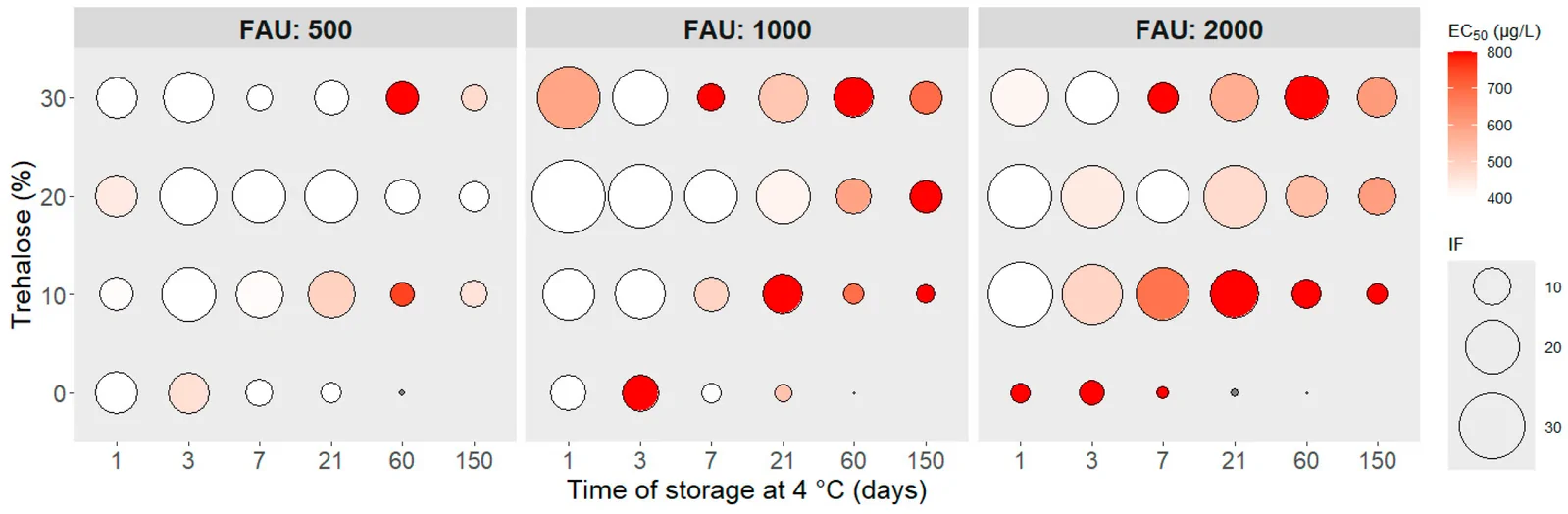
Long-term biosensor performance following storage at different cell densities (500, 1000 and 2000 FAU) and trehalose concentrations (0%, 10%, 20%, 30%). EC_50_ (in µg/L) and max IF values of the freeze-dried RYAs are shown against the storage time. Multiple plates with the stated conditions were prepared and stored at 4 °C for 1, 3, 7, 21, 60 and 150 days after freeze-drying. Cells were reconstituted and induced with T4. Abs_578_ was measured after an exposure time of 2 h. The IF values were calculated, and four-parameter sigmoidal functions were fitted to the dose–response data of each condition (n = 2) to determine the EC_50_ and LOD values (see Table S2). EC_50_ values of 800 µg/L and above are displayed in red.

In accordance with the results displayed in Figure 1, cell concentrations of 500, 1000 and 2000 FAU were selected for investigating the effect of long-term storage on biosensor stability and performance. Data was collected after 1, 3, 7, 21, 60 and 150 days of storage of the lyophilized samples at 4 °C, and the results are shown in Figure 3. Despite the declined performance previously observed at cell densities higher than 1000 FAU, this condition was still included in the long-term experiments, since a higher initial cell density may compensate for loss of cell viability during storage. In accordance with the initial screening experiment (Figure 1), it becomes apparent that, especially for the cell concentrations of 1000 and 2000 FAU, biosensor performance without a cryoprotectant is already poor right after freeze-drying. Although the cell density of 500 FAU in the absence of trehalose resulted in storability on the timescale of weeks, the IF dropped below 3.5 after 21 days of storage. Upon stabilization with 10% trehalose, the biosensor performance improved throughout all tested cell densities, with the highest benefit for high cell densities, but the storability did not exceed the timescale of weeks (1000 and 2000 FAU), or the biosensor performance was unstable (500 FAU). The overall best results were achieved with the addition of 20% trehalose. While the IF values were not substantially higher when compared to the results obtained with 10% trehalose, the EC_50_ remained more stable over the tested period. Activities at cell densities of 1000 and 2000 FAU declined after 150 days, but the tested cell density of 500 FAU yielded stable results up to 5 months. Regarding the latter, the EC_50_ of 332 ± 54 µg/L and LOD of 28 ± 11 µg/L are virtually similar to the detection sensitivity observed directly after freeze-drying (EC_50_ = 447 ± 60 µg/L, LOD = 34 ± 9 µg/L) and are in the same order of magnitude as the assay results using fresh yeast cells (171 ± 13 µg/L and 13 ± 3 µg/L). A further increase in trehalose concentration to 30% did not further improve the biosensor performance. Like the results obtained with 10% trehalose, the biosensor performance was unstable after a time span of 7 days, sometimes yielding reasonable results (500 FAU, 150 days) but often failing completely even at early timepoints (1000 FAU or 2000 FAU, 7 days).

Whenever the biosensor performed well (EC_50_ for T4 < 500 µg/L), the obtained LOD values were in the range of 10 to 55 µg/L and thus in the same order of magnitude as the result obtained with fresh yeast (13 µg/L). Only if the EC_50_ exceeded the 500 µg/L range, LODs of 60 to 200 µg/L were observed (Table S2).

### 3.4. Effect of Long-Term Storage on Cell Survival

**Figure 4:**
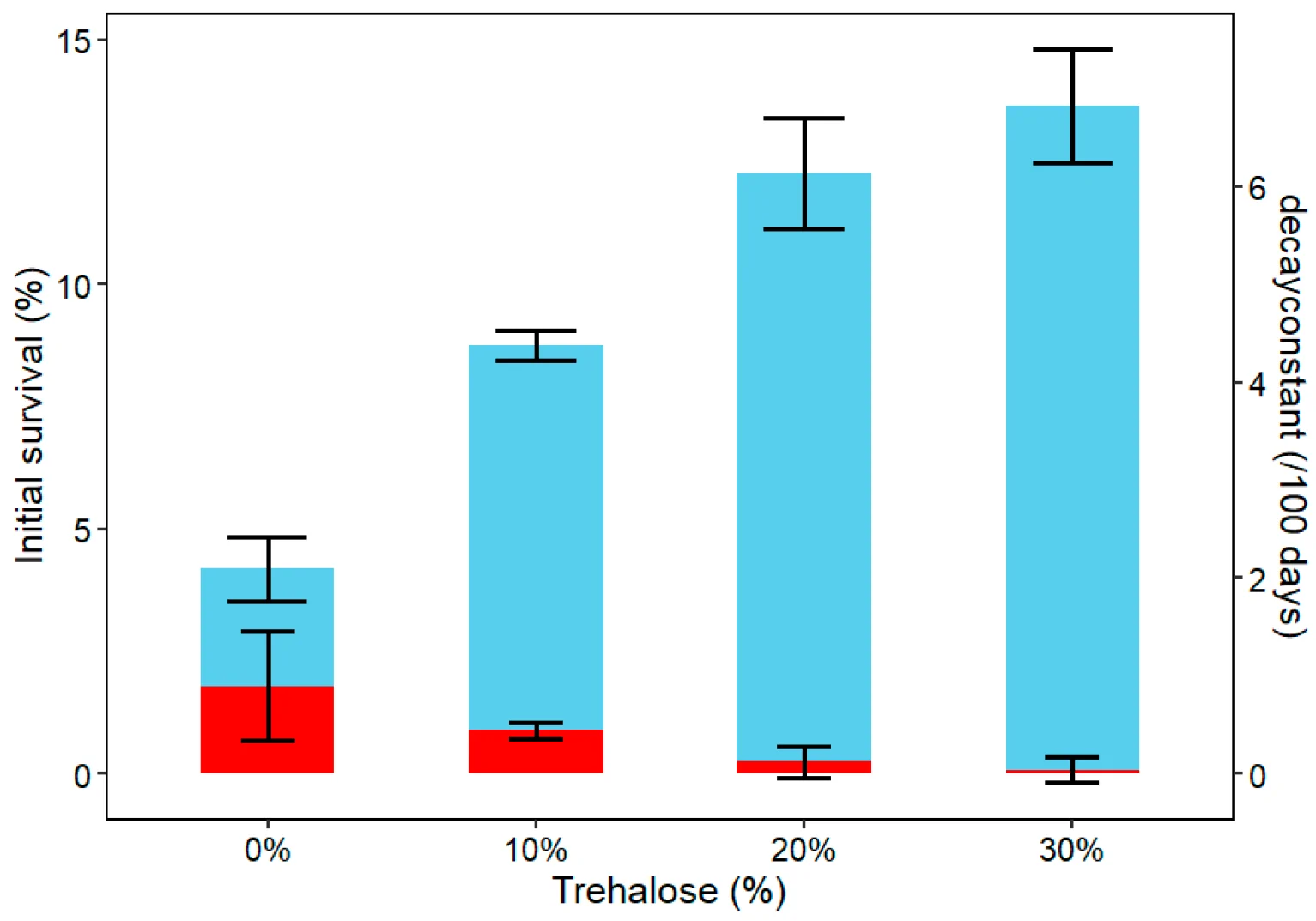
Survival rate of the biosensor cells after the freeze-drying process (blue) and follow-up decay per 100 days (red) at different trehalose concentrations. The CFUs of the three cell densities used (500, 1000 and 2000 FAU) were determined prior to freeze-drying (n = 3) and compared against the CFUs of the corresponding cell suspension at different storage times (1, 3, 7, 21, 60 and 150 days, n = 2). The results are expressed as the fraction of CFUs observed after the freeze-drying process compared to the CFUs of the same condition before freeze-drying. Due to the high variability, the results of the three cell densities (see Table S3b) are combined. Exponential decay models are fitted, and the resulting initial survival rate (blue), as well as the decay constant per 100 days (red), is presented.

To quantify the impact of the freeze-drying conditions on cell viability, the rate of cell survival was monitored in parallel to the activity assays. Survival was determined by counting the number of CFUs compared to the same cell suspension prior to freeze-drying. To improve the data basis for statistical analysis, survival rates of different cell densities were combined and are displayed only as a function of cryoprotectant concentration. Absolute values are given in the SI. A model of exponential decay was fitted to the data of each trehalose concentration tested, resulting in values for the initial survival as well as the corresponding decay constant (Figure 4). Without a cryoprotectant, only 4 ± 1% of cells survived the freeze-drying process, with a decay constant of 1 ± 1 for every 100 days of storage at 4 °C. With addition of 10% trehalose, the initial cell survival rose significantly to 9 ± 1% (*p* < 0.01), with a reduced decay constant of 0.4 ± 0.1 per 100 days of storage. Upon increasing the trehalose concentration to 20%, the initial cell survival rose significantly to 12.3 ± 1.1% (*p* < 0.01) with a decay constant of 0.1 ± 0.2 per 100 days of storage. The highest stabilization is detected upon incubation with 30% trehalose, where a survival rate of 13.6 ± 1.2% with a decay constant of 0.0 ± 0.1 per 100 days of storage was observed. However, this improvement in cell viability is not significant when compared to the results obtained with 20% trehalose (*p* = 0.22). Based on the decay model, it takes 167 days (10% trehalose), 912 (20% trehalose) or even 3222 days (30% trehalose) for the stabilized yeast cells to reach the survival rate of 4% observed for the unstabilized cells.

### 3.5. Effect of Medium Viscosity on Biosensor Performance

**Figure 5:**
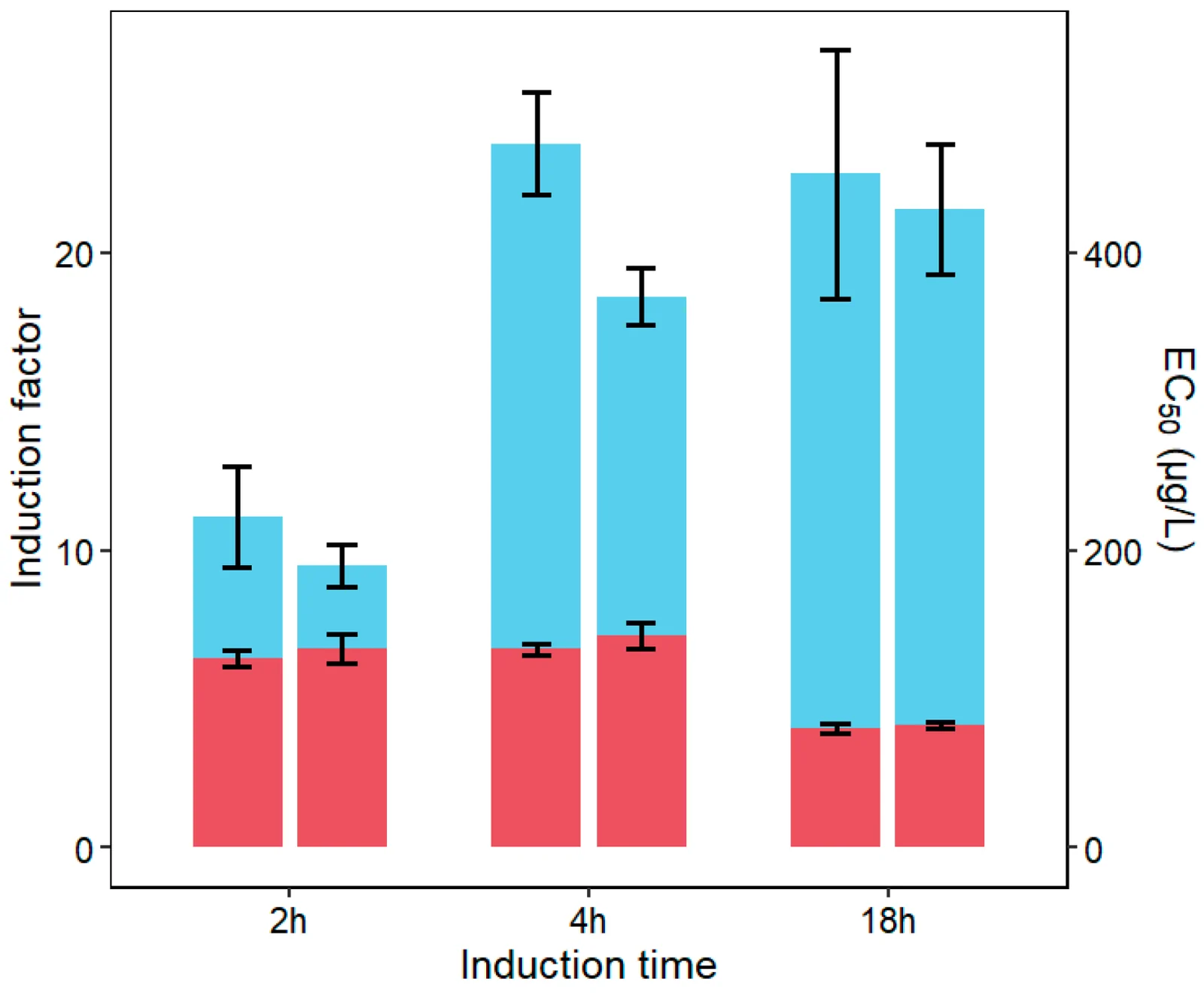
IF (blue) and EC_50_ (red) of freshly prepared yeast assays without (−) and with (+) trehalose after induction times of 2, 4 and 18 h. Cell suspensions with 150 FAU and optional 30% trehalose (*w*/*v*) were exposed to T4. Abs_578_ was measured, and the IF values were calculated (n = 2). Four-parameter sigmoidal functions were fitted, from which the EC_50_ values were determined. Error bars show the absolute error.

Interestingly, while the cell survival was highest using 30% trehalose, thyroxine detection performance of the resuscitated cells was optimal at 20% trehalose. To test the hypothesis that an increased medium viscosity, caused by the addition of concentrated trehalose solution, does change the biosensor performance, fresh RYAs were conducted. This excludes the effects of freeze-drying, since a possible viscosity effect would also be observable when using fresh cells. The assays were conducted using a cell density of 150 FAU because, due to cell growth, this cell density corresponds to the cell density of 500–1000 FAU used for freeze-drying. Activity measurements were performed after exposure times of 2, 4 and 18 h in the presence and absence of a 30% trehalose addition (Figure 5).

Regarding the EC_50_, values of 128 ± 6 µg/L, 134 ± 4 µg/L and 81 ± 3 µg/L were determined for the standard assay, and values of 134 ± 10 µg/L, 143 ± 9 µg/L and 83 ± 2 µg/L were determined for the assay incubated with 30% trehalose after the timespans of 2, 4 and 18 h. The maximum biosensor induction increased from 11.1 to 23.7 and then decreased to 22.6 for the fresh assay, while for the assays incubated with trehalose, induction values of 9.5, 18.5 and 21.5 were measured. With an increase in incubation time to 18 h, the EC_50_ decreases significantly (*p* < 0.01) when compared to incubation times of 2 and 4 h, unaffected by the presence of trehalose. When the EC_50_ values of both assays are compared against each other after any of the mentioned timepoints, no significant deviation between the results obtained with and without trehalose can be observed. However, a significant difference (*p* < 0.01) between results with and without trehalose can be observed regarding the biosensor induction after an incubation time of 4 h.

### 3.6. Testing the Freeze-Dried RYA with Real Environmental Matrices

**Figure 6:**
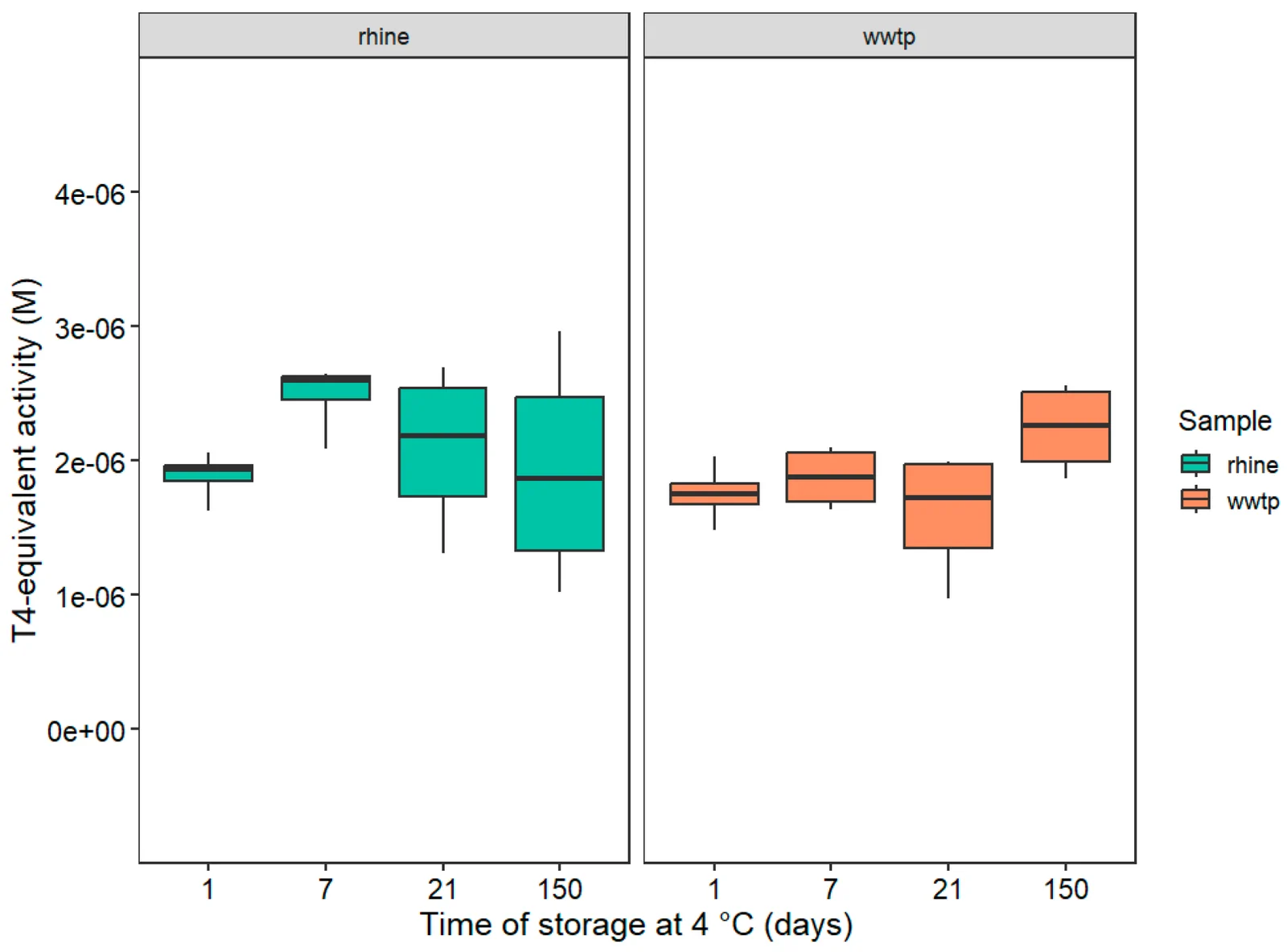
Performance of the freeze-dried RYAs upon induction with spiked river water and spiked WWTP extract (2 µM T4) after storage times of 1, 7, 21 and 150 days. Freeze-dried cells (1000 FAU, 20% trehalose) were reconstituted and exposed to the spiked samples. Abs_578_ was measured, and the IF values were calculated (n = 2). Four-parameter sigmoidal functions were fitted to the reference dose–response data, and four T4-equivalent sample concentrations (see Table S4) were calculated per condition based on T4-dilution steps 3 to 6. The box plot shows the median, upper and lower quartiles and highest and lowest values for the calculated T4-equivalent concentrations.

In parallel to the characterization of the concentration–response curve of the reference compound T4, spiked environmental samples were tested to challenge the freeze-dried yeast cells with realistic sample matrices. For this purpose, a native surface water and a concentrated wastewater effluent were spiked to a final concentration of 2 µM T4. Since these plates had to be prepared in line with the plates prepared for the long-term experiments, the cell concentration of 1000 FAU and trehalose concentration of 20% were chosen based on the results from Section 3.1. Plates were exposed to the two spiked environmental samples after storage times of 1, 7, 21 and 150 days. The results are shown in Figure 6.

The freeze-dried RYAs detected T4-equivalent activities of 1.9, 2.5, 2.1 and 1.9 µM in the spiked Rhine water and 1.8, 1.9, 1.6 and 2.3 µM of T4-equivalent activities in the spiked wastewater effluent after the four storage periods listed above. While the variability between each of the four obtained T4-equivalent activities increased over time, no systematic divergence of the biosensor results was detected over the tested timeframe for the environmental samples tested.

## 4. Discussion

With an increasing demand for the on-site application of microbial biosensors, different stabilization and immobilization methods have emerged. While most reports focused on matrix embedding, this approach does not fully solve the storability problem since storage time is usually reported in the timescale of 4 to 6 weeks [23,34,35]. In the present study, we report a successful yeast thyroid screen by a freeze-dried biosensor stored for up to 5 months, with performance comparable to a freshly prepared control. The benefits of the proposed method lie not only in allowing institutions with limited lab access to perform RYAs but also in its amenability to field use. One of these applications is portable biosensor arrays, which require stabilized cells that are storable and usable in lab-free conditions. To keep cost and complexity to a minimum, a single cryoprotectant was employed: trehalose, the efficiency of which as a cryoprotectant was previously documented [27,29]. Indeed, we demonstrate here that the addition of trehalose successfully stabilized the cells during the freeze-drying process. The maximal induction intensity in response to thyroxine dropped by a range of 2- to 6-fold, and the detection sensitivity, namely EC_50_ and LOD values, dropped by 1.5- to 3-fold when compared to freshly prepared cells. While both effects were significant, the observed 1.5- to 3-fold loss in sensitivity can be compensated for during sample preparation or sample enrichment. Due to the harsh conditions of the freeze-drying process, high cell mortality is to be expected. The initial survival rate of 4.2% for unstabilized cells determined in the current study is in accordance with earlier reports [24]. This observation is consistent with the fact that during the pre-screening test, optimal cell densities were obtained when the cell density was increased about 20-fold compared to the cell density of 25 to 100 FAU that is commonly used for fresh RYAs. Without any stabilization, the freeze-dried cells also showed the highest decay constant during storage, leading to poor results in the long-term experiments. Interestingly, the lowest tested cell density of 500 FAU demonstrated a reasonable performance for up to 3 weeks even without the addition of trehalose. This may be counterintuitive, since a low initial cell density also results in a low absolute number of viable cells after freeze-drying, but a low cell density and no addition of trehalose are the best conditions to facilitate complete dehydration during the freeze-drying process due to the low viscosity of the cell suspension. It has to be pointed out that the prevention of rehydration is the key to satisfactory storability [27,36]. While all plates were successfully vacuum-sealed in plastic bags, a full vacuum cannot be reached by the device used, and residual water traces may still remain after the freeze-drying process [36]. Premature rehydration can be suppressed further by storing the plates under freezing conditions. Under these conditions, usability of up to 10 months has been reported [27]. In all of the conducted experiments in this study, the addition of a cryoprotectant has been beneficial when compared to the unstabilized cells. One of the main benefits is the increase in the maximum biosensor induction, indicating improved cell viability when a cryoprotectant is present. This trehalose-dependent increase was observed along the entire duration of the long-term storage experiment. The observation that trehalose increases cell viability is also supported by the number of CFUs. Here a 2-fold increase in cell survival from 4.2% to 8.7% (and higher) could be observed after the freeze-drying process when compared to cells freeze-dried without a cryoprotectant. The decay constant was also reduced by a factor of 2 by the addition of 10% trehalose. However, an addition of 10% trehalose did not stabilize the cells sufficiently after a timespan of weeks, especially for higher cell densities. This may be linked to the problem discussed above, where a high cell density impedes complete dehydration of the cells during the freeze-drying process due to an increase in solution viscosity. With the addition of 20% trehalose, the maximum biosensor induction increased slightly and stabilization for up to 5 months could be observed, with a substantial reduction in the decay constant. Here the increase in cryoprotectant concentration again resulted in increased initial cell survival—in this case to over 12%—connected to a further reduction in cell decay. Regarding the pre-test it is worth mentioning that the test did perform well in indicating conditions suitable for long-term stabilization, although the test performance was determined by the z-factor right after the freeze-drying process instead of the loss of sensitivity over time. Both the best condition (500 FAU, 20%, z-factor of 0.77) and the worst condition out of the other cell densities tested (2000 FAU, 0%, z-factor of 0.06) did match the results of the long-term experiments. Furthermore, it becomes apparent that a high z-factor correlates better than the IF with the observed high long-term stability. While a high IF is important to yield a well-performing biosensor, the increase in IF when cell density is increased from 500 to 1000 FAU, for example, does not result in better biosensor performance. The test showed no further improvement with an increase in trehalose concentration to 30%. This observation fits with the results of the long-term storage experiments. When discussing the optimal concentration of cryoprotectant, two opposing trends need to be considered: On the one hand, the cell viability and long-term survival rate seem to increase with the addition of trehalose, where addition even beyond 20% showed a further reduction in the decay rate and an improvement in initial cell viability. On the other hand, an increase in trehalose and cell concentration also increases the viscosity of the solution. At 30 °C the viscosity of pure water is around 0.80 cP [37]. A 20% trehalose solution has a viscosity of about 2 cP, and a 30% trehalose solution has a viscosity of around 3 cP [38]. Based on the Stokes–Einstein equation, this results in a reduction in the thyroxin diffusion coefficient by about 75% in a 30% trehalose solution compared to water. However, as shown here, this trehalose concentration did not affect detection sensitivity of RYAs performed with fresh cells. The only notable difference was that the biosensor induction lagged behind the trehalose-free assay if trehalose was added. This is true for the incubation times of 2 and 4 h, before values for biosensor induction reached a maximum after 18 h. Thus, the addition of trehalose, increasing the viscosity of the cell suspension, may partially explain the observed reduction in the IF but cannot explain the observed reduction in sensitivity after freeze-drying.

In a recent study the impact of freeze-drying on the intracellular and extracellular metabolite profiles of *Saccharomyces cerevisiae* was studied using high-resolution LC-MS/MS-based metabolomics [39]. Interestingly, a depletion of intracellular levels of the essential amino acids proline and glutamic acid was found. Considering these limitations a delayed expression of intracellular proteins is likely to also affect the synthesis of the reporter enzyme β-galactosidase. In the present study, the revitalization of the yeast cells was performed for 2 h before the addition of the sample. Eventually, this time span may not be sufficient to fully replenish the intracellular amino acid pool. A prolonged revitalization might compensate for this effect but would result in an increased time requirement for the method. The study suggests the supplementation of proline and glutamic acid before freeze-drying to improve viability and the metabolic capacity of the resuscitated cells.

As noted above, viscous solutions take longer to be fully dehydrated, which may increase the amount of residual water and thus contribute to premature and reduced storability. In the case of 20% trehalose and a cell density of 1000 FAU, the plates of the long-term experiments tested after 150 days may not have been sealed properly, since the decrease in biosensor performance does not fit the observations for both other cell densities at 500 and 2000 FAU and the corresponding long-term experiment with environmental matrices after the same time period of 150 days. Here, the tested conditions resulted in a stable biosensor, yielding accurate results even after 5 months. While the variability in the case of the Rhine water sample results increased after 5 months, the obtained mean T4-equivalent activities are very close to the spiked amount of 2 µM of T4 while shortening the multi-day sterile process to only 5 h in a nonsterile environment, demonstrating the principle of a freeze-dried biosensor.

Since a total of twelve different conditions were tested regarding the long-term experiments, due to feasibility reasons, only two independent replicates (n = 2) could be included in this study. In addition, the long-term experiments regarding the environmental matrices were conducted using relatively high concentrations of T4, while environmentally relevant concentrations are much lower. This study demonstrates the proof of principle of a freeze-dried biosensor, but due to these limitations, a full characterization and additional experiments are necessary before the biosensor is actually field-deployable. Future work needs to include the preparation of more independent replicates to further assess the reliability and reproducibility. Furthermore, the assay performance needs to be evaluated at near-LOD concentrations to investigate the biosensor response at environmentally relevant concentrations. In addition, the statements regarding the effect of residual water on the biosensor performance and storability need to be addressed. This includes a physicochemical characterization of the bioassay under each condition, tracking residual moisture and connecting the results to the observed behavior to further understand, explain and improve the variability of the freeze-dried assay.

This study demonstrates the proof of principle of this freeze-dried biosensor and promotes the generation of a ready-to-use, lab-independent biosensor with on-site applicability, providing fast and reliable detection of EDCs in environmental samples without the need for highly specialized staff and equipment. With respect to the application of the envisaged device in the field, regulations concerning the use of genetically modified microorganisms (GMMs) have to be considered. Since the GMMs used in the described procedure are contained and not released, permission for their use for field measurements is likely.

## Data Availability

The raw data supporting the conclusions of this article will be made public by the authors.

## Supplementary Materials

The following supporting information can be downloaded at https://www.mdpi.com/article/10.3390/bios16080441/s1, Figure S1: Calibration curve of the Tecan Photometer for FAU calculation. Table S1: The IF and z-factor as a function of cell density and concentration of cryoprotectant. Table S2: Long-time biosensor performance following long-term storage for 1, 3, 7, 21, 60 and 150 days after freeze-drying at different cell densities (500, 1000 and 2000 FAU) and trehalose concentrations (0%, 10%, 20%, 30%). Table S3: (a) Counted absolute CFUs for the fresh cell suspensions in triplicates; (b) Counted absolute CFUs of the three cell densities used (500, 1000 and 2000 FAU) as a function of the concentration of cryoprotectant (0%, 10%, 20%, 30%) and storage time (1, 3, 7, 21, 60 and 150 days, n = 2). Table S4: Performance of the freeze-dried RYAs upon induction with spiked river water and spiked WWTP extract (2 µM T4) after storage times of 1, 7, 21 and 150 days at 4 °C.
