# Supplementary material for: A Ready-to-Use Recombinant Yeast Two-Hybrid Assay for Thyroxine Detection
Source: Biosensors (Basel). 2026 Aug 15;16(8):441. doi: 10.3390/bios16080441 (PMC13510673; doi:10.3390/bios16080441)
Supplement: Supplementary file 1 [file biosensors-16-00441-s001.zip › biosensors-4439152-supplementary.pdf]

Supplementary Information

A ready-to-use recombinant yeast two-hybrid assay for thyroxine detection

Marius Danhausen [1], Sebastian Buchinger [1,*], Shimshon Belkin [2], Thomas A. Ternes [1]

1 Federal Institute of Hydrology (BfG), Am Mainzer Tor 1, 56068 Koblenz

2 The Alexander Silberman Institute of Life Sciences, The Hebrew University of Jerusalem, Edmond J. Safra Campus - Givat Ram, Jerusalem 9190401, Israel; shimshon.belkin@mail.huji.ac.il

** Correspondence: buchinger@bafg.de; +49 261 13065316*

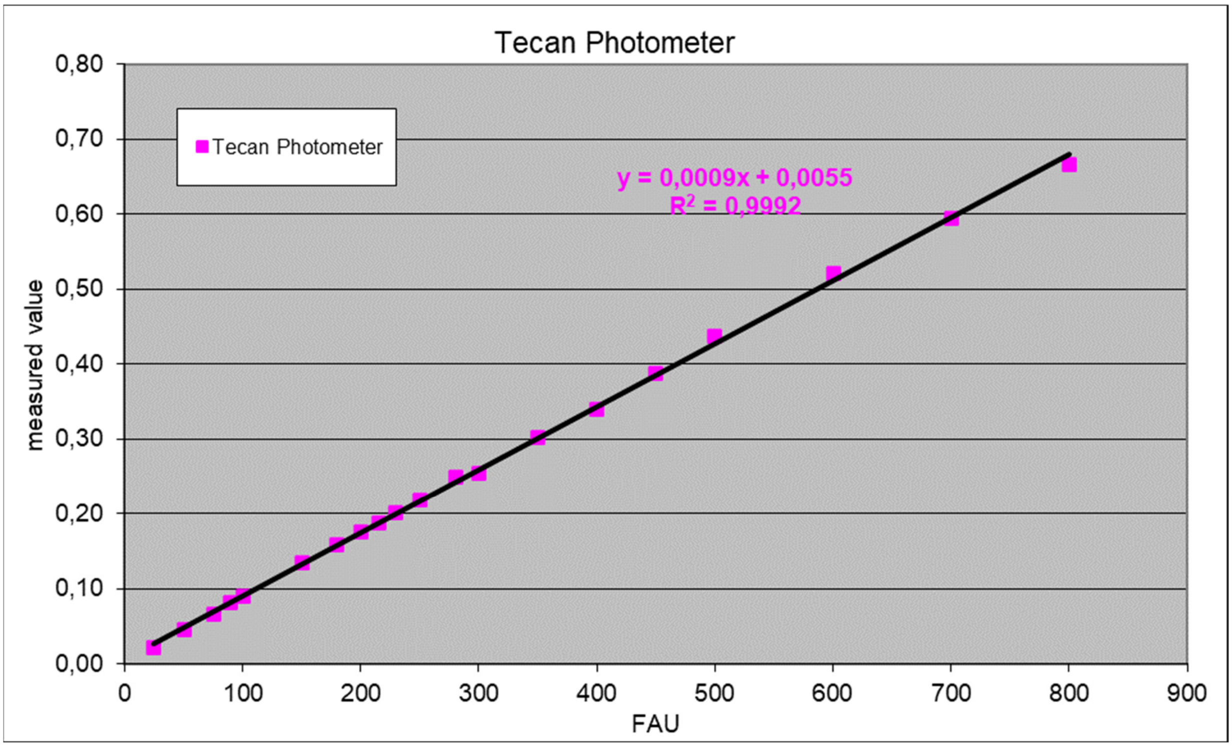

Figure S1. Calibration-curve of the Tecan Photometer for FAU calculation. A solution of 5 g hexamethylenetetramine ($C_6H_{12}N_4$) and 0.5 g hydrazine sulfate ($N_2H_6SO_4$) in 100 mL ddH_2O corresponds to a 4000 FAU stock solution. Out of this solution, a dilution series is prepared ranging from 800 to 25 FAU and absorption measurements are performed at 600 nm (Abs_{600}). The obtained values are plotted against the FAU of the corresponding solution.

Table S1. The IF and z-factor in dependence of cell density and concentration of cryoprotectant. The cells were directly reconstituted after freeze-drying and induced with 1 µL of 0.25 µM T4.

Trehalose-concentration	FAU	Induction-factor	z-factor
30%	25	1,92	0,21
20%	25	2,21	0,49
10%	25	2,00	0,19
0%	25	1,32	-0,59
30%	50	3,12	0,52
20%	50	3,21	0,56

10%	50	2,61	0,24
0%	50	1,59	-0,27
30%	100	4,85	0,54
20%	100	5,08	0,50
10%	100	4,19	0,47
0%	100	2,17	-0,05
30%	150	6,14	0,57
20%	150	6,38	0,53
10%	150	5,42	0,39
0%	150	3,01	0,22
30%	250	8,50	0,23
20%	250	9,38	0,60
10%	250	7,61	0,36
0%	250	4,02	0,22
30%	350	7,62	0,17
20%	350	10,92	0,60
10%	350	9,31	0,60
0%	350	4,57	0,53
30%	500	12,55	0,54
20%	500	14,28	0,77
10%	500	11,09	0,49
0%	500	6,06	0,58
30%	1000	14,10	0,56
20%	1000	15,70	0,68
10%	1000	12,49	0,57
0%	1000	7,49	0,36
30%	2000	8,61	0,47

20%	2000	7,98	0,29
10%	2000	8,52	0,40
0%	2000	4,63	0,06
30%	4000	2,81	-0,23
20%	4000	3,04	-0,36
10%	4000	2,23	-0,94
0%	4000	1,43	-0,99

Table S2. Long-time biosensor performance following long-term storage for 1, 3, 7, 21, 60 and 150 days after freeze-drying at different cell densities (500, 1000 and 2000 FAU) and trehalose concentrations (0%, 10%, 20%, 30%). EC_{50}, LOD and max IF values of the freeze-dried RYAs are shown. The IF values were calculated and four-parametric sigmoidal functions were fitted to the dose-response data of each condition (n=2) to determine the EC_{50} and LOD values.

Trehalose-concentration	FAU	storage time [d]	EC_{50} [µg/L]	Induction-factor	LOD [µg/L]
0	500	1	196,48	12,11	10,65
10	500	1	406,61	7,96	25,14
20	500	1	446,63	12,37	33,71
30	500	1	391,67	11,91	24,55
0	500	3	466,31	11,98	12,12
10	500	3	297,98	19,79	24,56
20	500	3	294,86	22,50	27,93
30	500	3	227,19	17,84	24,66
0	500	7	345,76	5,38	14,84
10	500	7	409,33	15,51	20,74
20	500	7	308,97	18,88	35,40
30	500	7	397,83	5,11	30,32

0	500	21	237,51	3,49	17,02
10	500	21	497,15	15,33	n.d.
20	500	21	300,82	19,39	22,82
30	500	21	324,18	8,62	60,30
0	500	60	n.d.	1,14	n.d.
10	500	60	752,31	4,38	32,78
20	500	60	396,05	8,33	17,72
30	500	60	1457,79	7,29	40,61
0	500	150	n.d.	n.d.	n.d.
10	500	150	462,25	5,31	81,06
20	500	150	332,17	6,72	28,21
30	500	150	478,45	5,09	n.d.
0	1000	1	315,04	9,17	14,04
10	1000	1	368,88	18,19	23,85
20	1000	1	382,58	36,35	24,37
30	1000	1	589,92	26,27	28,81
0	1000	3	915,07	9,67	21,47
10	1000	3	365,38	17,79	34,22
20	1000	3	349,31	28,07	35,00
30	1000	3	280,67	20,57	33,55
0	1000	7	257,26	3,29	22,36
10	1000	7	491,36	8,68	23,20
20	1000	7	338,91	19,35	55,25
30	1000	7	801,88	5,56	34,56
0	1000	21	526,58	2,61	42,03
10	1000	21	1036,72	11,07	33,60
20	1000	21	424,48	19,61	28,06

30	1000	21	521,13	16,72	33,02
0	1000	60	2466,87	1,05	n.d.
10	1000	60	689,86	3,55	187,66
20	1000	60	594,36	8,90	19,63
30	1000	60	1256,76	11,11	36,45
0	1000	150	n.d.	n.d.	n.d.
10	1000	150	3526,12	2,97	109,24
20	1000	150	893,72	7,59	24,98
30	1000	150	693,94	7,32	90,45
0	2000	1	2052,79	3,06	34,69
10	2000	1	375,95	28,97	30,97
20	2000	1	381,35	28,01	24,06
30	2000	1	418,20	22,08	32,52
0	2000	3	1656,44	4,81	53,30
10	2000	3	493,01	25,14	52,77
20	2000	3	443,12	26,91	55,35
30	2000	3	337,66	19,01	27,48
0	2000	7	8124,61	1,70	154,27
10	2000	7	682,50	19,38	96,65
20	2000	7	340,08	18,87	57,37
30	2000	7	798,22	6,80	51,05
0	2000	21	290163,20	1,31	161,70
10	2000	21	1676,41	16,30	92,53
20	2000	21	479,93	26,33	54,44
30	2000	21	573,24	16,39	68,90
0	2000	60	4652,65	1,06	n.d.
10	2000	60	1572,32	6,08	191,36

20	2000	60	538,35	12,23	23,34
30	2000	60	1629,18	13,85	130,49
0	2000	150	n.d.	n.d.	n.d.
10	2000	150	949,14	3,56	601,70
20	2000	150	604,25	9,75	32,18
30	2000	150	609,28	10,89	180,77

Table S3a. Counted absolute CFUs for the fresh cell suspensions in triplicates.

500 FAU	1000 FAU	2000 FAU
8	17	17
4	5	38
1	15	27

Table S3b. Counted absolute CFUs of the three cell densities used (500, 1000 and 2000 FAU) in dependence of the concentration of cryoprotectant (0%, 10%, 20%, 30%) and storage time (1, 3, 7, 21, 60 and 150 days, n=2). Due to the high variability, the results of the three cell densities are combined during data procession.

	days of storage	*1*	*3*	*7*	*21*	*60*	*150*
500 FAU	0%	**5**	**9**	**9**	**7**	**2**	**0**
	0%	**7**	**3**	**4**	**3**	**4**	**3**
	mean	6	6	6,5	5	3	1,5
	survival-rate	5,54%	5,54%	6,00%	4,62%	2,77%	1,38%
	10%	**13**	**5**	**20**	**13**	**6**	**1**
	10%	**8**	**19**	**12**	**12**	**10**	**4**

	mean	10,5	12	16	12,5	8	2,5
	survival-rate	9,69%	11,08%	14,77%	11,54%	7,38%	2,31%
	20%	**18**	**17**	**17**	**19**	**21**	**12**
	20%	**27**	**9**	**19**	**7**	**11**	**8**
	mean	22,5	13	18	13	16	10
	survival-rate	20,77%	12,00%	16,62%	12,00%	14,77%	9,23%
	30%	**21**	**11**	**25**	**15**	**5**	**20**
	30%	**20**	**28**	**8**	**12**	**30**	**19**
	mean	20,5	19,5	16,5	13,5	17,5	19,5
	survival-rate	18,92%	18,00%	15,23%	12,46%	16,15%	18,00%
1000 FAU	0%	**18**	**1**	**1**	**0**	**2**	**5**
	0%	**5**	**8**	**8**	**3**	**8**	**0**
	mean	11,5	4,5	4,5	1,5	5	2,5
	survival-rate	3,73%	1,46%	1,46%	0,49%	1,62%	0,81%
	10%	**13**	**18**	**35**	**20**	**35**	**30**
	10%	**24**	**36**	**18**	**18**	**21**	**17**
	mean	18,5	27	26,5	19	28	23,5
	survival-rate	6,00%	8,76%	8,59%	6,16%	9,08%	7,62%
	20%	**43**	**43**	**18**	**59**	**39**	**60**
	20%	**63**	**22**	**30**	**47**	**45**	**20**
	mean	53	32,5	24	53	42	40

	survival-rate	17,19%	10,54%	7,78%	17,19%	13,62%	12,97%
	30%	**69**	**68**	**21**	**36**	**63**	**51**
	30%	**40**	**36**	**37**	**48**	**39**	**19**
	mean	54,5	52	29	42	51	35
	survival-rate	17,68%	16,86%	9,41%	13,62%	16,54%	11,35%
2000 FAU	0%	**57**	**20**	**40**	**28**	**16**	**27**
	0%	**59**	**9**	**11**	**12**	**22**	**7**
	mean	58	14,5	25,5	20	19	17
	survival-rate	8,49%	2,12%	3,73%	2,93%	2,78%	2,49%
	10%	**48**	**16**	**44**	**65**	**20**	**11**
	10%	**68**	**53**	**11**	**42**	**27**	**33**
	mean	58	34,5	27,5	53,5	23,5	22
	survival-rate	8,49%	5,05%	4,02%	7,83%	3,44%	3,22%
	20%	**54**	**43**	**44**	**30**	**84**	**78**
	20%	**63**	**83**	**34**	**51**	**42**	**27**
	mean	58,5	63	39	40,5	63	52,5
	survival-rate	8,56%	9,22%	5,71%	5,93%	9,22%	7,68%
	30%	**61**	**78**	**74**	**53**	**74**	**61**
	30%	**84**	**64**	**55**	**39**	**86**	**61**
	mean	72,5	71	64,5	46	80	61

	survival-rate	10,61%	10,39%	9,44%	6,73%	11,71%	8,93%

Table S4. Performance of the freeze-dried-RYAs upon induction with spiked river water and spiked WWTP extract (2 µM T4) after storage times of 1, 7, 21 and 150 days at 4 °C. Freeze-dried cells (1000 FAU, 20% trehalose) were reconstituted and exposed to spiked Rhine water or spiked WWTP extract both resulting in a max. well concentration of 2 µM. Four-parametric sigmoidal functions were fitted to the reference dose-response data and four T4-equivalent sample concentrations were calculated per condition based on T4-dilution steps 3 to 6.

T4-equiv. [µM]	sample	Storage-time in days
1,937	rhine	1
2,054	rhine	1
1,926	rhine	1
1,623	rhine	1
1,758	wwtp	1
2,030	wwtp	1
1,735	wwtp	1
1,482	wwtp	1
2,618	rhine	7
2,572	rhine	7
2,642	rhine	7
2,088	rhine	7
2,042	wwtp	7
2,102	wwtp	7
1,632	wwtp	7
1,713	wwtp	7

2,691	rhine	21
2,493	rhine	21
1,873	rhine	21
1,306	rhine	21
1,992	wwtp	21
1,967	wwtp	21
1,474	wwtp	21
0,976	wwtp	21
2,965	rhine	150
2,305	rhine	150
1,426	rhine	150
1,016	rhine	150
2,563	wwtp	150
2,026	wwtp	150
1,870	wwtp	150
2,499	wwtp	150
